# PuraMatrix hydrogel enhances the expression of motor neuron progenitor marker and improves adhesion and proliferation of motor neuron-like cells 

**DOI:** 10.22038/ijbms.2020.39797.9434

**Published:** 2020-04

**Authors:** Marzieh Darvishi, Hatef Ghasemi Hamidabadi, Sajad Sahab Negah, Ardeshir Moayeri, Taki Tiraihi, Javad Mirnajafi-Zadeh, Ali Jahanbazi Jahan-Abad, Amir Shojaei

**Affiliations:** 1 Department of Anatomy, Faculty of Medicine, Ilam University of Medical Sciences, Ilam, Iran; 2 Shefa Neuroscience Research Center, Khatam Alanbia Hospital, Tehran, Iran; 3 Department of Anatomy & Cell Biology, Faculty of Medicine, Mazandaran University of Medical Sciences, Sari, Iran; 4 Immunogenetic Research Center, Department of Anatomy & Cell Biology, Faculty of Medicine, Mazandaran University of Medical Sciences, Sari, Iran; 5 Neuroscience Research Center, Mashhad University of Medical Sciences, Mashhad, Iran; 6 Department of Anatomical Sciences, Faculty of Medical Sciences, School of Medical Sciences, Tarbiat Modares University, Tehran, Iran; 7 Department of Physiology, Faculty of Medical Sciences, Tarbiat Modares University, Tehran, Iran; 8 Department of Brain and Cognitive Sciences, Cell Science Research Center, Royan Institute for Stem Cell Biology and Technology, ACECR, Tehran, Iran

**Keywords:** Motor neuron-like cells, Nanoscaffolds, Proliferation, Stem cell therapy, Three-dimension culture, Tissue engineering

## Abstract

**Objective(s):**

Cell therapy has provided clinical applications to the treatment of motor neuron diseases. The current obstacle in stem cell therapy is to direct differentiation of stem cells into neurons in the neurodegenerative disorders. Biomaterial scaffolds can improve cell differentiation and are widely used in translational medicine and tissue engineering. The aim of this study was to compare the efficiency of two-dimensional with a three-dimensional culture system in their ability to generate functional motor neuron-like cells from adipose-derived stem cells.

**Materials and Methods:**

We compared motor neuron-like cells derived from rat adipose tissue in differentiation, adhesion, proliferation, and functional properties on two-dimensional with three-dimensional culture systems. Neural differentiation was analyzed by immunocytochemistry for immature (Islet1) and mature (HB9, ChAT, and synaptophysin) motor neuron markers.

**Results:**

Our results indicated that the three-dimensional environment exhibited an increase in the number of Islet1. In contrast, two-dimensional culture system resulted in more homeobox gene (HB9), Choline Acetyltransferase (ChAT), and synaptophysin positive cells. The results of this investigation showed that proliferation and adhesion of motor neuron-like cells significantly increased in three-dimensional compared with two-dimensional environments.

**Conclusion:**

The findings of this study suggested that three-dimension may create a proliferative niche for motor neuron-like cells. Overall, this study strengthens the idea that three-dimensional culture may mimic neural stem cell environment for neural tissue regeneration.

## Introduction

In recent years, neural tissue engineering has emerged as a promising treatment for central nervous system (CNS) regeneration (1, 2). The strategy of tissue engineering is replacement of lost cells and manipulation of the environment of the damaged tissue to facilitate axon regeneration (2). However, one of the major obstacles to the success of neural tissue engineering is the low efficiency to generate functional neurons from grafted cells. Establishment of methods to enhance neuronal differentiation from stem cells, such as neural stem cells (NSCs), adipose derived stem cells (ADSCs), induced pluripotent stem cells (iPSCs), and embryonic stem cells, is important for disease modeling, drug screening, and cell transplantation therapy for neurodegenerative diseases (3, 4). Differentiation of stem cells into motor neurons is considered the most promising therapeutic strategy for replacing degenerated cells in the brain injuries including traumatic brain injury (TBI), spinal cord injury (SCI), amyotrophic lateral sclerosis (ALS), and stroke (4–6). 

 NSCs have self-renewal ability and can differentiate into neurons, astrocytes, or oligodendrocytes. NSCs, a promising cell type for replacement therapy, are known to be effective for treatment in animal models of neurodegenerative diseases and injuries such as TBI, SCI, and stroke where neuronal cells are reduced. NSCs can also be produced from adult sources by direct trans-differentiation of cells cultured from adult tissue into NSCs. Some of these sources include adult adipose tissue, bone marrow, and skin cells. NSCs derived from ADSCs under the neurosphere method have a great potential to differentiate into functional motor neurons (7). 

Extensive research has shown that stem cells are highly sensitive to their environment (3). A novel scheme for differentiation of stem cells is to provide a 3D environment in order to mimic the extracellular matrix (ECM)-(8). Biomaterial scaffolds can be used as a platform for cells to improve the differentiation of stem cells (9,10). During the past decades, several biocompatible materials have been considered to stimulate nerve regeneration. Hydrogel is a 3D scaffold which is shown to exhibit the physical and chemical properties of the extracellular matrix and is suitable for transplantation *in vivo* (11,12). Among different types of hydrogels, BD™ PuraMatrix™ peptide hydrogel (PM), or RADA16, is a synthetic matrix that spontaneously self-assembles into β-sheets structures to make a 3D environment similar to the ECM (13). Recently investigators have demonstrated that PM supports the differentiation of NSCs into neurons (14). Therefore, expandable sources of motor neuron-like cells and ideal scaffolds are needed for successful CNS repair (15). The aim of this study was to investigate the differentiation capacity of NSCs derived from ADSCs into motor neuron like cells (MNLCs) in a 2D and 3D environment at the presence of growth factors. To confirm the effect of PM on differentiation, a comparison has been performed with the 2D culture system. The differentiation, proliferation, viability, and electrophysiological properties of cells were evaluated. 

## Materials and Methods

***Isolation of ADSCs***

Adult female Sprague–Dawley (SD) rats (200–250 g) were provided by the animal facility of the Razi Institute, Tehran, Iran. The animals were housed under adequate ventilation with free access to food and water at room temperature (22±2 ^°^C). All animal protocols were approved by the Ethical Committee on Animal Experimentation of the Ilam University of Medical Sciences, Ilam, Iran. The ADSCs was isolated from perinephric fat tissue as previously described (16). Briefly, the fat tissue was minced, washed extensively to remove contaminating hematopoietic cells, the tissue fragments were incubated with 0.075% collagenase type I (Sigma Company) and DMEM (Dulbecco’s Modified Eagle Medium) for 30 min at 37 ^°^C, and then neutralized through 10% FBS (Fetal Bovine Serum) into DMEM (Gibcobrl, Eggenstein, Germany) and centrifuged (1000 RPM) for 10 min. The cellular deposition was incubated and cultured in a T25 flask containing DMEM with 10% FBS, 100 U/ml penicillin, and 100 μg streptomycin for four to six days at 37 ^°^C. The cells were harvested through trypsin/EDTA (0.05% trypsin/ 0.5 mM EDTA: GIBCO-BRL, Eggenstein, Germany) and cultured for four passages. 

***Induction of neural stem cell (NSCs) from ADSCs***

 To induce NSCs, ADSCs were cultured in Dulbecco’s modified Eagle’s medium (DMEM)/F-12 containing 2% B-27 supplement, 20 ng/ml epidermal growth factor (EGF), and 10 ng/ml basic fibroblast growth factor (bFGF)-(Invitrogen, Paisley, Scotland). Neurospheres were formed after seven days. 

***Differentiate NSCs into MNLCs***

Differentiation of NSCs to MNLCs was performed as describe previously (13, 16). The NSCs were seeded in a 24-well plate coated with poly L-lysine at 1×10^5^ cell/ml density (50% confluency) and incubated in DMEM/F12 medium with Sonic hedgehog ( Shh; 1 ug/ml), retinoic acid (0.1 M), EGF (20 ng/mL), bFGF (20 ng/ml), and B27 (1%). For differentiation into motor neurons, media were changed to neurobasal medium containing Neurotrophin-3 (NT-3; 5 ng/ml), brain derived neurotrophic factor (BDNF; 10 ng/ml), glial derived neurotrophic factor (GDNF; 10 ng/ml), and ciliary neurotrophic factor (CNTF; 5 ng/ml) for five days. 

***Whole-cell patch clamp recording***

To assess functional differentiation, electrophysiological recordings of cells were used at the end of the induction protocol. The differentiated neurons were seeded on poly-l-lysine-coated coverslips. After incubation of the cells at room temperature for 30 min, they were transferred into a submerged recording chamber continually perfused at 1.5–2.5 ml/min with standard artificial cerebrospinal fluid (ACSF) at room temperature (23–25 ^°^C). The ACSF was constantly bubbled with 95% O2–5% CO_2_ and contained (in mM) 125 NaCl, 1.25 NaH_2_PO_4_, 3 KCl, 10 D-glucose, 25 NaHCO_3_, 1.3 MgCl2, and 2 CaCl_2_. The recording chamber was mounted on a fixed-stage upright microscope (Axioskop 2 FS MOT; Carl Zeiss, Göttingen, Germany). An IR-CCD camera (IR-1000, MTI, USA) with a ×40 water immersion objective lens for cell detection was used. Patch pipettes were pulled from borosilicate glass (1.5-mm outer diameter; Harvard Apparatus, Edenbridge, UK) using a horizontal puller (P-97, Sutter Instrument, Novato, CA, USA). The intracellular solution consists of 115 K-gluconate, 10 HEPES, 20 KCl, 10 disodium-phosphocreatine, 2 EGTA, 0.3 NaGTP, and 2 MgATP was used for filling the pipettes. The pH was adjusted to 7.25–7.30 and osmolality to 285– 290 milliosmoles. The electrode tip resistance in the bath was typically 5–7 MΩ and the series resistance ranged from 10 to 30 MΩ. The data were low-pass filtered at 10 kHz and acquired at 10 kHz with a Multiclamp 700B amplifier equipped with a Digi-data 1440 A/D converter (Molecular Devices, Sunnyvale, CA, USA). The signal was recorded on a PC using Axon pClamp 10 acquisition software (Molecular Devices, Palo Alto, CA) (17). 

***Preparation of scaffold***

PM was purchased from BD Biosciences, USA. Purity and identities of the peptides were confirmed by analytical high-performance liquid chromatography. All aqueous peptide solution was prepared by using Milli-Q water (18.2 MΩ), stored at 4 ^°^C, and sonicated for 30 min before use. The volume of 1% PM was prepared by dilution in deionized water containing 20% sucrose. In the 3D group, 50 μl of 0.15% PM was seeded to the 96-well plate. 

***Immunocytochemistry***

To assess cell phenotype and differentiation potential, immunocytochemistry was performed. Cells were rinsed with PBS and fixed in 4% paraformaldehyde at room temperature for 30 min. Samples were incubated in the permeation solution (Triton X-100 0.3%, 25 min) and blocked with FBS 5%. Cells were immunostained at 4 ^°^C overnight with different primary antibodies including CD105 (fat-derived mesenchymal stem cell marker), CD90 (mesenchymal stem cells marker), CD49d (specific marker for fat cells), CD31 (an endothelial cells marker), CD106 (a marker of mesenchymal stem cells derived from bone marrow stromal cells), and CD45 (a hematopoietic stem cells marker) (Table 1). The antibodies to anti-nestin, anti-NT-68, and anti-Sox2 were used to label the undifferentiated NSCs. To confirm differentiation to MNLCs, the antibodies to islet-1 as a motor neuron precursor’s marker and homeobox gene (HB9), Choline Acetyltransferase (ChAT), and synaptophysin as the markers for mature neural cell types were used. The cells were then incubated with secondary antibodies for 1 hour at room temperature (Table 1). After washes, the nuclei were stained with Propidium iodide (PI) and visualized with a fluorescent microscope (Olympus IX71: Olympus, Japan). The negative control was performed by incubating the cells with a secondary antibody without the presence of the primary antibody.

***Functional cell evaluation***

In order to evaluate the function of the cells, FM1-43 (synaptic vesicle release), RH795 (determination of action potential with voltage-sensitive dye), and Fluo-4 NW (calcium ion shift) methods were used.

***Co-culture***

 To determine the functionality of MNLCs, they were co-cultured with myotubes derived from mouse muscle cell lines (C2C12 myoblasts, American Type Culture Collection: Rockville, Md, USA) (21, 22). The cell lines (C2C12) were cultured with DMEM containing 10% FBS (Invitrogen*, *Paisley, Scotland) on cover slips. The myotube formation was achieved using 2% horse serum (Invitrogen*, *Paisley, Scotland) for three to seven days. Five days after differentiation, cells were used for co-culture with the MNLCs.

***FM1-43 staining***

 In order to evaluate the synaptic vesicle release in the MNLCs, FM1-43 staining was used (18). The efficiency of FM1-43 (Molecular Probes, Leiden, The Netherlands) staining was assessed using an inverted Epifluorescent microscope (Olympus IX71: Olympus, Japan). The MNLCs were seeded in 24-well plates at 5×10^3^ density (2D and 3D culture systems); after washing, these cells were exposed to saline containing 170 mM NaCl, 3.5 mM KCl, 0.4 mM KH_2_PO_4_, 5 mM NaHCO_3_,1.2 mM Na_2_SO_4_, 1.2 mM MgCl_2_, 1.3 mM CaCl_2_, 5 mM glucose, and 20 mM N tris(hydroxymethyl)-methyl-2-aminoethanesulfonic acid for 10 min. For the next step, the solution was replaced with stimulating Solution-1 containing saline supplement with 100 mMKCl and 10 μM FM1-43. After 2 min, this solution was changed to stimulating Solution-2 containing 3.5 mM KCl and 10 μM FM1-43. Next, the cells were exposed to the solution for 3 min. This performance was repeated three times. For unloading of dye, the solution was changed to saline with 100 mM KCl. The dye unloading was visualized using an inverted Epifluorescent microscope and photos were taken for 10 min with one photograph per min.

***Evaluation of calcium ion shift***

Transmission of intracellular Ca^2+^ in MNLCs was investigated by a fluorescent Ca^2+^ indicator (Fluo-4 NW: Molecular Probes, Leiden, The Netherlands) and followed by stimulation (16). The MNLCs were cultured in 24-well plates (2D and 3D culture systems) with medium containing 5% fetal calf serum for three to four days. The medium was discarded and 0.5 ml of the dye loading solution (Fura-2 solution (1 mM)) was added to each well plate. The plates were incubated at 37 ^°^C for 20 min and removed from the Fura 2-AM and washed with HEPES (4-(2-hydroxyethyl)-1-piperazineethanesulfonic acid) buffer saline and left for 1 hr in the HEPES buffer saline. 

***Determination of action potential with RH 795***

The MNLCs were seeded in 24-well plates at 5×10^3^ density with and without PM (16). The culture medium was removed from the MNLCs and then 20 µl of the RH 795 loading solution (4 mM) with ACSF was added to the MNLCs. These cells were washed 3 times and incubated for one hr in ACSF buffer under dark conditions and the RH 795 unloading was visualized using an inverted Epifluorescent microscope with settings for excitation at 380 nm (calcium free) and 340 nm (calcium complex) with fixed emission at 510 nm (10 min with one photograph per min).

***Cell Adhesion***

For cell adhesion assays, MNLCs were cultured on hydrogel PM scaffold as a 3D culture and 2D plastic plate culture at a density of 2×10^4^ cells in 24-well plates. Initial cell adhesion was assessed at 2 hr after incubation by inverting the culture plate to remove non-adhered cells. The adhered cells were then fixed using 4% paraformaldehyde in PBS and then the nuclei were stained using DAPI. Fluorescence images were taken at several spots on each surface and the adhered cells were counted using particle analysis in ImageJ. 

***Proliferation assay***

To assess cell proliferation, MTS-based cell proliferation assay (CellTiter 96® AQueous One Solution, Promega, USA) was used. 2×10^4^ cells/well were seeded in scaffold and cultured at days 1, 7, and 14. Culture medium (80 μl) was discarded and added to 20-μl MTS solution for 3 hr at 37 ^°^C. A medium without cells was used as blank solution. The optical density value was measured at 490 nm by microplate reader (Biotek, USA). Three parallel replicates were read for each sample.

***Statistical analysis***

Analyses were performed using SPSS version 16 software (SPSS Inc., Chicago, USA). Statistically significant differences between groups were assessed by One-way analysis of variance (ANOVA). *Post-hoc* multiple comparisons were performed using Tukey’s tests. All data were presented as mean±standard deviation (SD) and significance level was regarded at *P*<0.05.

## Results

***Rat ADSCs isolation***

**Figure 1:**
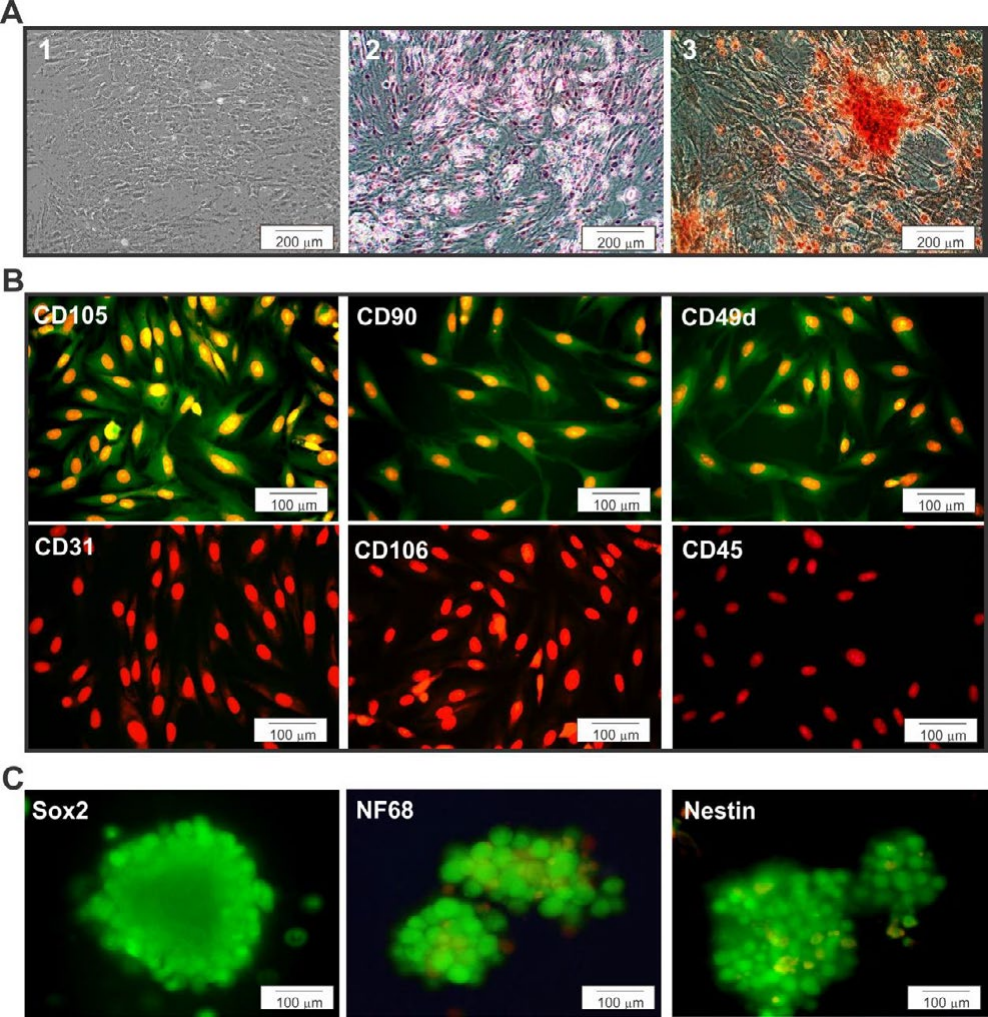
Differentiation capacity of ADSCs was assessed by specific markers. A-1) Phase contrast images of the third passage of the ADSCs culture. A-2, 3) Lipogenic and osteogenic differentiation of ADSCs stained with Oil red and Alizarin red stain, respectively. Images of differentiated adipogenic and osteogenic cultures showing phenotypic changes as well as lipid droplet accumulation and mineralization of the cultures. B) ADSCs were labeled with primary antibodies; CD105, CD90, CD49d, CD31, CD106, and CD45. Immunocytochemistry showing positive staining for CD105, CD90, and CD49d markers used to confirm ADSCs stemness. C) Immunocytochemistry of neural stem cell markers (SOX2, NT68, and Nestin) was performed. Markers are shown in green, while the cell nuclei, counterstained with propidium iodide (PI), are shown in red

**Figure 2:**
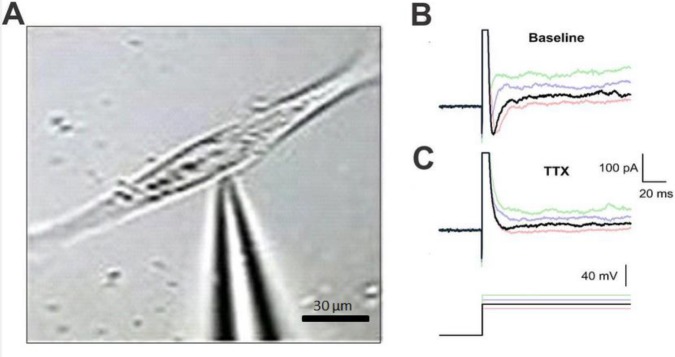
Electrophysiological properties of MNLCs at the end of induction assessed by whole-cell patch clamp recording. A) Phase contrast image of a patch pipette attached to the membrane of a cultured MNLCs. Representative traces of membrane potential in response to 200, 400, and 600 as the pale traces and 800 pA as the dark trace in depolarizing current (660 ms) before (B) and after (C) 1 μM of tetrodotoxin (TTX) treatment. MNLCs at the end of induction (n=15) showed single action potential like spikes. Treatment with 1 μM of TTX as a blocker of voltage-gated Na+ channels inhibited spike firing confirming sodium action potential-like event in these cells

**Figure 3.**
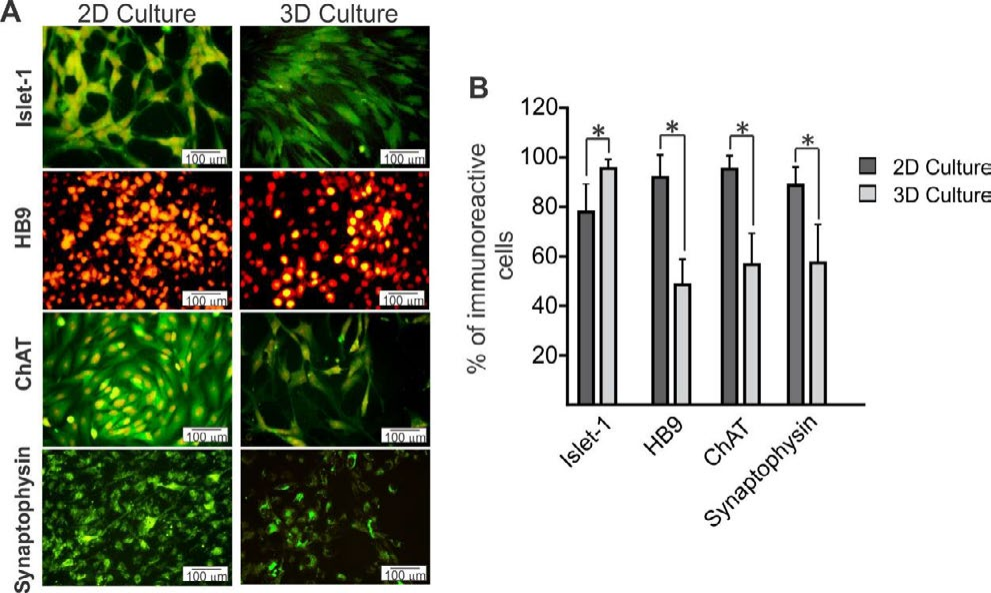
Immunofluorescence studies were performed to investigate the expression of the motor neuron markers in two different groups. A) MNLCs were immunostained with primary antibodies; islet-1, HB9, ChAT, and synaptophysin. Markers are shown in green and the cell nuclei (counterstained with PI) are shown in red. B) Quantitative data of positive cells in 2D and 3D groups. Our results showed that islet-1 significantly increased in 3D compared with the 2D group. The higher expression of HB9, ChAT, and synaptophysin was observed in 2D more than in 3D. Data are presented as mean±SD. *P<0.05

After three passages, the ADSCs showed a homogeneous composition in monolayer with fibroblast-like morphology (Figure 1A-1). The differentiation of ADSCs into mesenchymal lineages such as lipogenic and osteogenic phenotypes has been shown in Figure 1. The results showed that lipogenic differentiation had cell morphology with lipid accumulation in the form of small vacuoles or droplets, which were stained with Oil Red (Figure 1A-2). Osteoblast-like cells were capable of mineralizing extracellular matrix and staining with Alizarin Red dye (Figure 1A-3). 

ADSCs were stained with CD90, CD105, and CD49d as positive makers and CD106, CD31, and CD45 as negative markers (Figure 1B). Immunocytochemistry results showed that over 95% of ADSCs expressed CD90, CD105, and CD49d. The results also showed that ADSCs were negative for immunofluorescence staining with CD34, CD45, and CD106 (Figure 1B). 

***Induced NSCs derived from ADSCs***

To confirm the induction of ADSCs into NSCs, immunocytochemistry was performed. The results, as shown in Figure 1C, indicate that the majority of cells expressed NSCs markers such as SOX2, NT68, and nestin. 

***Functional differentiation of MNLCs from NSCs***

We assessed the functionality of the MNLCs-derived from NSCs. To evaluate the functionality of induced neurons, patch clamp recording at the end of the differentiation process was performed (Figure 2A). Neuron-like cells (n=15) were used at resting membrane and action potentials. To check for changes in membrane voltage following injection of depolarizing and hyperpolarizing currents, the current clamp mode was used. The recorded resting membrane varied between −20 and −30 mV. Before current injection, voltage was adjusted to −65 mV (Figure 2B). A single spike was observed following current injection in the induced neurons, but not repetitive spike firing. The maximum amplitude of spikes was ~40 mV (Figure 2). The most interesting aspect of these data are preliminary electrophysiological properties of MNLCs *in vitro*. From the data in Figure 2C, it is apparent that after pretreatment with TTX (1 μM) as a sodium channel blocker, the firing of repetitive spikes disappeared, which has implications for the presence of sodium currents in neuron-like cells at the end of the induction protocol.

***The expression of motor neuron markers***

To compare the expression of motor neuron markers between 2D and 3D groups, immunostaining of islet-1, HB9, ChAT, and synaptophysin was performed (Figure 3A). Our results showed that islet1 as an immature motor neuron marker significantly increased in the 3D group compared with the 2D group (Figure 3B; *P*<0.05). In contrast to the expression of islet-1, mature motor neuron markers such as HB9, ChAT, and synaptophysin significantly increased in the 2D group compared with the 3D group (Figure 3B; *P*<0.05). 

***Co-culture***

**Figure 4:**
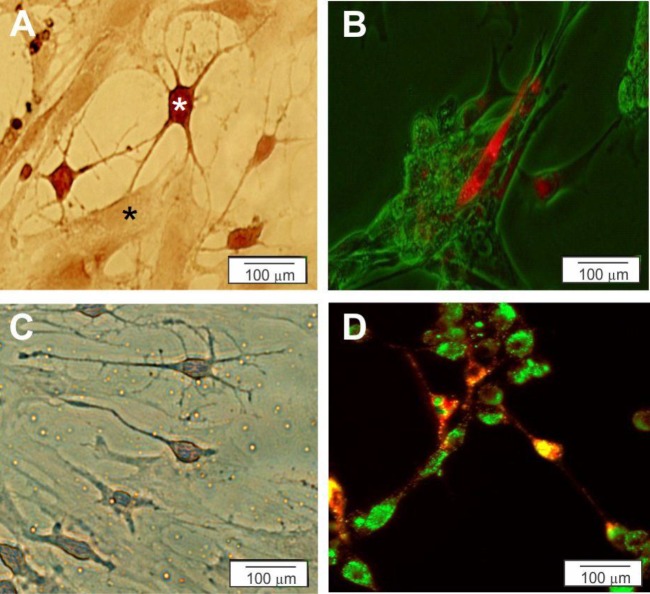
Characterization of the MNLCs was assessed by co-culturing with myotubes (C2C12) on 2D (A and B) and 3D (C and D) culture plates. A and C) Myotubes co-cultured with MNLCs was stained by Cresyl violet on 2D (A) and 3D (C) culture plates (black star is Myotubes and white star is MNLCs). B and D) Myotubes were stained with PKh67 (green) and co-cultured with the MNLCs were stained with PKh26 (red) on 2D (B) and 3D (D) culture plates

Phase contrast images of MNLCs showed an extensive network of processes with myotubes for both 2D- (Figure 4A) and 3D-culture systems (Figure 4C). To detect the synaptic region, PKh67 staining was also performed. MNLCs connected to the myotubes in both 2D- (Figure 4B) and 3D culture systems (Figure 4D). 

***FM1-43 staining***

**Figure 5:**
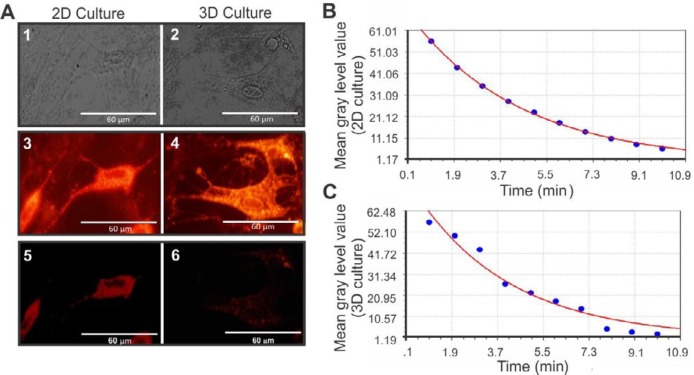
Assessment of the synaptic vesicles of the MNLCs seeded in 3D and 2D culture were performed by FM1-43 staining. A) The phase contrast image of the MNLCs cultured on 2D and 3D culture plate. A fluorescence image of the same field in A1-5, photographed after 1 min and 10 min following the de-staining on 2D plates. A4-6 were also photographed after 1 min and 10 min following the de-staining of MNLCs on 3D culture plate. B and C represent the curve of the monoexponential decay model for the time points following de-staining of MNLCs culture on 2D (y= 80.7e^-0.265x^ ) and 3D (y=70.4e^-0.231x^ ) systems, respectively

To determine the synaptic vesicles (SVs), MNLCs were stained with the fluorescent membrane marker FM1-43 (Figure 5A). To evaluate the secretion rate of SVs, a monoexponential decay model was used. The secretion rate in the MNLCs were observed in both 2D culture plates (Figure 5B) (the equation , standard error= 4.41, and correlation coefficient= 0.978) and 3D culture systems (Figure 5C) (the equation , standard error= 0.59, and correlation coefficient= 0.99). 

***Calcium ion shift***

**Figure 6:**
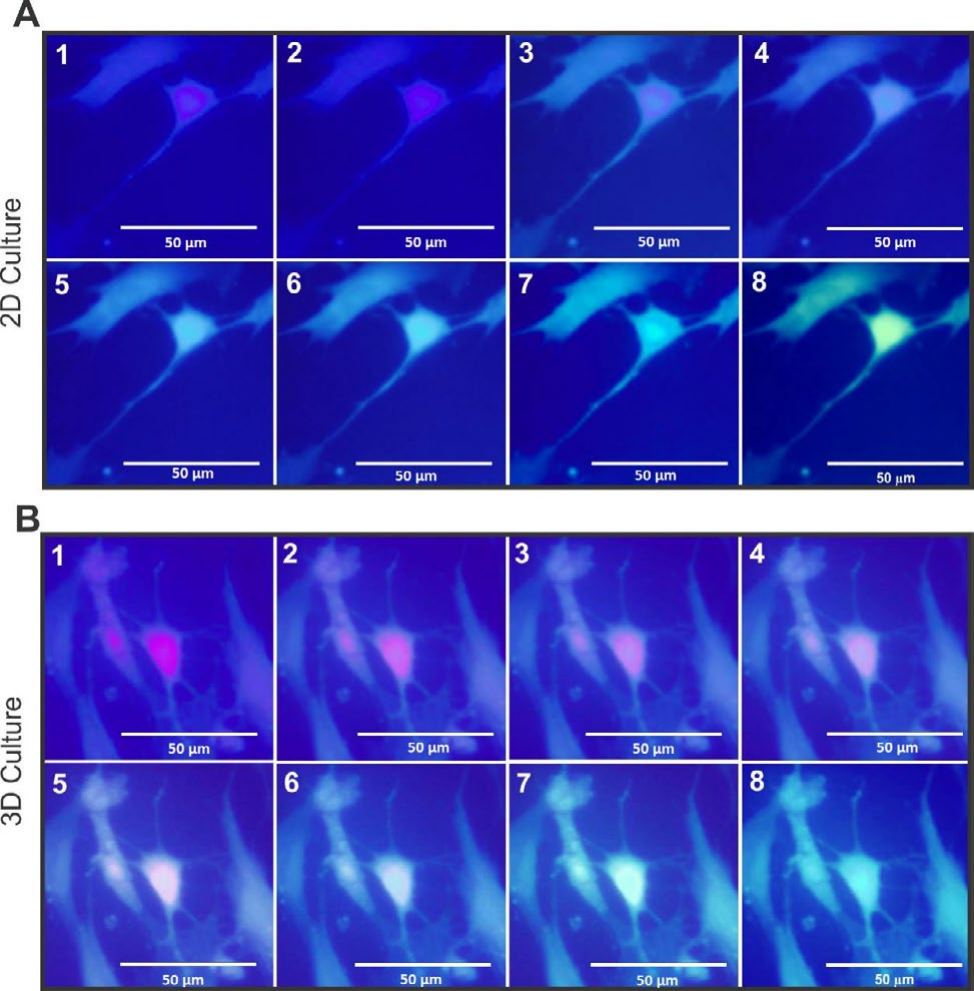
The Ca^2+^ concentration of MNLCs was detected by Fluo-4 NW assay. A represents the MNLCs stained with Fluo-4 NW followed by their stimulation on 2D culture plate and B on 3D culture. Both groups of serial image show changes in the color of the cells following a shift in the intracellular Ca^2+^

The staining of the differentiated MNLCs with Ca^2+^ indicator (Fluo-4 NW) has been presented in Figure 6, where MNLCs showed changes in the color depending on the intracellular Ca^2+^ concentration. The transfer of Ca^2+^ were assessed in 2D (Figure 6A) and 3D (Figure 6B) culture systems. 

***Voltage-sensitive dye (VSD) imaging***

**Figure 7:**
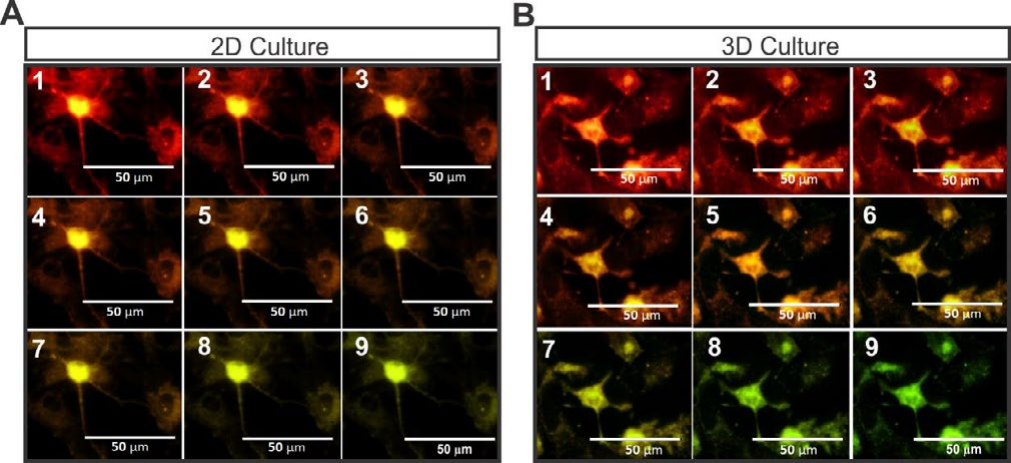
The staining of the MNLCs with voltage-sensitive dye (RH795) followed by their stimulation on 2D and 3D culture systems. A shows the membrane depolarization and repolarization of the MNLCs with change of color in 2D, and B represents the same field photographed serially demonstrated membrane action potential with same mechanism on 3D culture plates

Figure 7 shows the action potential changes in MNLCs membranes by shift of color from red to green. The red color depicts membrane depolarization and the green color shows the membrane repolarization. In this method, the membrane performs simultaneous monitoring of several cells 2D (Figure 7A) and 3D (Figure 7B) culture systems. 

***Cell attachment***

**Figure 8:**
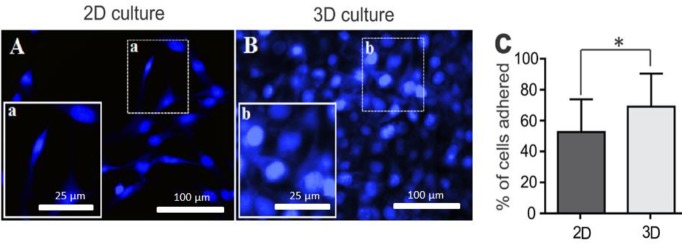
Initial cell attachment was evaluated by DAPI stain at 2 hr post-plating. A and B represent immunofluorescent staining of two groups. C shows the quantitative data of adherent cells. The average percentages were calculated using five different views. Data are shown as mean±SD. *P<0.05

MNLCs were cultivated on 2D (Figure 8A) and 3D (Figure 8B) cultures for a period of 2 hr to detect the amount of initial cell adhesion. The percentage of cell adhesion on 3D surfaces was significantly higher than that of the 2D-culture group (Figure 8C; *P*<0.05).

***Proliferation assay***

**Figure 9:**
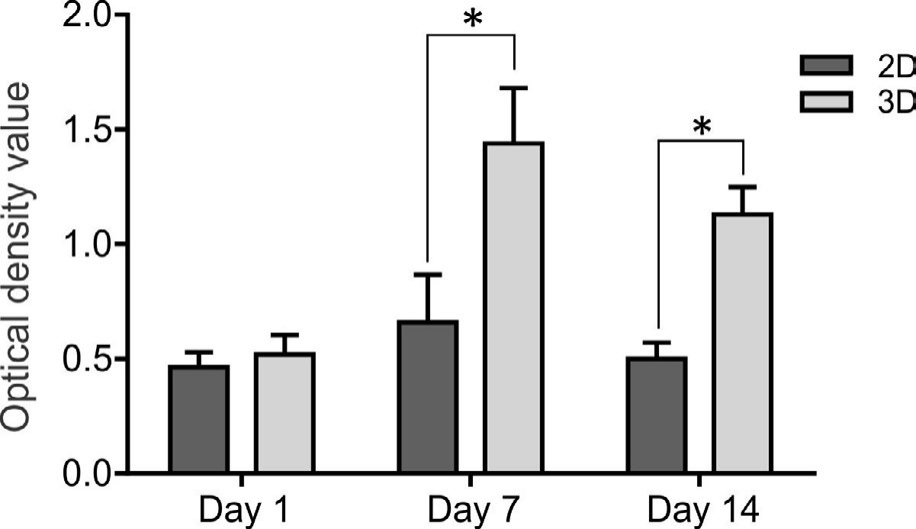
Proliferation of MNLCs was detected by MTS assay on days 1, 7, and 14. The proliferation assay showed significant difference between groups in 2D and 3D cultures. Data are shown as mean±SD. *P<0.05

The results on the first day did not show significant differences between the two media. These results showed that cell proliferation in the PM scaffold was significantly higher compared with the 2D culture system on days 7 and 14 (Figure 9; *P*<0.05). These data confirmed the biocompatibility of PM scaffolds for proliferation and viability of the MNLCs. 

## Discussion

In this study, we performed a direct comparison of 2D and 3D culture systems to evaluate which of these might be more efficient in differentiation and functional behavior of motor neurons. We have generated neurons expressing multiple markers of immature and mature motor neurons from NSCs derived from ADSCs. Our results showed that an immature motor neuron marker had a high expression in 3D environment compared with the 2D culture system. On the other hand, mature motor neuron markers such as HB9, ChAT, and synaptophysin expressed more in 2D compared with 3D. Qualitative results revealed that MNLCs had the same behavior in terms of SVs, intracellular Ca^2+^ concentration, and membrane depolarization. We also compared the proliferation and adhesion of MNLCs in 2D and 3D. MNLCs adhesion and proliferation improved when embedded in the 3D environment compared with 2D. 

There is evidence that stem cell therapy is one of the most promising approaches for the treatment of ALS.  Motor neurons derived from human, rodent, and primate embryonic stem cells as well as iPSCs and murine cortical stem cells survived and extended *in vitro* and *in vivo* (4, 5, 19–21). In our study, NSCs derived from ADSCs and differentiated into motor neurons. Achieving the best results in stem cell therapy, depends on improved cell survival and differentiation-desirable cells. It has been demonstrated that cell-matrix adhesion plays a crucial role in cell proliferation, differentiation, and viability (22). Previous studies have shown that 3D scaffolds can enhance the differentiation and proliferation of stem cells (23, 24). A variety of scaffolds have been used for this purpose. PM is a hydrogel self-assembled by natural amino acids that can be modified with bioactive components to improve cell interactions (25, 26). In this study, we used PM because it has several advantages, including biocompatibility, nontoxicity, and no immunogenicity (24, 27). In addition, it has been shown that PM promoted adult mouse NSCs attachment, survival, proliferation, and differentiation (28).

Prior studies have noted the importance of the environmental cues on the fate of a specific cell type (29). For example, stiffness of the material surrounding mesenchymal stem cells (MSCs) is sufficient to direct them into the neuronal lineage (30). In order to analyze differences in neural fate in 2D- and 3D-culture systems, we studied Islet1 positive cell populations. Islet1 expresses in all postmitotic motor neurons and is required for various aspects of motor neurons development (31, 32). Based on our quantitative immunocytochemistry results, 3D generated more Islet1 positive cells, indicating progenitor neurons compared with the 2D culture. 

Next, we further analyzed the expression patterns of mature motor neuron markers such as ChAT, HB9, and synaptophysin in 2D and 3D culture systems. ChAT is the enzyme responsible for the synthesis of Ach (acetylcholine), which is expressed in high concentration in motor neurons (33-35). Our data showed a significant increase of ChAT positive cells in 2D compared with 3D. In addition, our results also indicated that 2D showed an increase in both HB9 and synaptophysin markers compared with 3D. Our results are in contrast with previous investigations suggesting that neural induction in 3D matrices increased the expression of mature neuron markers when compared with the 2D culture system (36, 37). This suggests that MNLCs require longer periods to reach maturation in the 3D culture condition.

We focused in more detail on the functional characteristics of MNLCs. We observed no significant differences between 2D and 3D environments in their SVs, intracellular Ca^2+^ concentration, and membrane depolarization, suggesting no profound changes in function was observed.

Finally, we evaluated the cell adhesion and proliferation in 2D and 3D groups. Our study showed that MNLCs proliferation was significantly improved in the 3D culture compared with 2D and provided a promising environment for cell adhesion. This agrees with the findings of previous studies that the 3D environment can be effective in increasing the cell survival (38). 

**Table 1:** Summary of primary antibodies used in immunocytochemistry

**Primary antibody **	**Cell target **	**Dilution **	**Host species**	** Company **	**Clonality**
**CD105**	fat-derived mesenchymal stem cell marker	1:200	Mouse	Millipore	Monoclonal
**CD49d **	specific marker for fat cells	1:300	Rabbit	Millipore	Polyclonal
**CD106 **	a marker of mesenchymal stem cells derived from bone marrow stromal cells	1:300	Mouse	Millipore	Monoclonal
**CD31**	an endothelial cells marker	1:300	Mouse	Millipore	Monoclonal
**CD45 **	a hematopoietic stem cells marker	1:300	Rabbit	Millipore	Polyclonal
**CD90 **	mesenchymal stem cells	1:300	Mouse	Millipore	Monoclonal
**Nestin **	Neural progenitor cells	1:100	Mouse	Millipore	Monoclonal
**NF68 **	Neuronal marker	1:200	Mouse	Millipore	Monoclonal
**Sox2 **	Neural stem cells marker	1:200	Rabbit	abcam	Polyclonal
**ISLET1**	Motor neuron like cells	1:200	Rabbit	abcam	Polyclonal
**HB9 **	Motor neuron like cells	1:200	Rabbit	abcam	Polyclonal
**Synaptophysin**	Mature neuron marker	1:200	Rabbit	abcam	Polyclonal
**ChAT**	Anti-Choline acetyltransferase maker	1:200	Mouse	Millipore	Monoclonal

The secondary antibodies: rabbit anti-mouse FITC-conjugated (R) or goat anti-rabbit FITC-conjugated (G) (abcam, Cambridge, UK); the titers of both secondary antibodies were 1:500

## Conclusion

This study has shown that NSCs-derived ADSCs were able to differentiate into motor neurons. The second major finding was that the expression of immature motor neuron marker significantly increased in the D culture compared with 2D. Taken together, these findings suggest a role for PM as a 3D environment in promoting a proliferative niche for MNLCs. Therefore, further investigations are needed to promote and guide neuronal differentiation by the 3D environment. 
